# Effects of user mental state on EEG-BCI performance

**DOI:** 10.3389/fnhum.2015.00308

**Published:** 2015-06-02

**Authors:** Andrew Myrden, Tom Chau

**Affiliations:** ^1^Holland Bloorview Kids Rehabilitation Hospital, Bloorview Research InstituteToronto, ON, Canada; ^2^Institute of Biomaterials and Biomedical Engineering, University of TorontoToronto, ON, Canada

**Keywords:** EEG, brain-computer interface, user mental state, fatigue, frustration, attention

## Abstract

Changes in psychological state have been proposed as a cause of variation in brain-computer interface performance, but little formal analysis has been conducted to support this hypothesis. In this study, we investigated the effects of three mental states—fatigue, frustration, and attention—on BCI performance. Twelve able-bodied participants were trained to use a two-class EEG-BCI based on the performance of user-specific mental tasks. Following training, participants completed three testing sessions, during which they used the BCI to play a simple maze navigation game while periodically reporting their perceived levels of fatigue, frustration, and attention. Statistical analysis indicated that there is a significant relationship between frustration and BCI performance while the relationship between fatigue and BCI performance approached significance. BCI performance was 7% lower than average when self-reported fatigue was low and 7% higher than average when self-reported frustration was moderate. A multivariate analysis of mental state revealed the presence of contiguous regions in mental state space where BCI performance was more accurate than average, suggesting the importance of moderate fatigue for achieving effortless focus on BCI control, frustration as a potential motivating factor, and attention as a compensatory mechanism to increasing frustration. Finally, a visual analysis showed the sensitivity of underlying class distributions to changes in mental state. Collectively, these results indicate that mental state is closely related to BCI performance, encouraging future development of psychologically adaptive BCIs.

## 1. Introduction

Brain-computer interfaces allow information to be conveyed to an external device, such as a computer, using cognitive activity alone (Mak and Wolpaw, 2009). Originally envisioned simply as a means of communication and environmental control for individuals with disabilities (Wolpaw et al., 2002), more and more prospective applications of BCIs have been proposed in recent years for both healthy and disabled individuals. BCIs have been harnessed for recreational purposes in gaming and virtual reality applications, where they provide an alternative input modality by which a simulation can be controlled (Lécuyer et al., 2008). BCIs have also been used to enable creative expression by translating cognitive activity into music and visual art (Miranda, 2006), to track changes in cognitive states such as alertness (Zander and Kothe, 2011), as a neurofeedback tool to achieve altered states of consciousness via meditation (Crowley et al., 2010), and in neurorehabilitation for individuals who have lost motor control due to stroke (Ang et al., 2010).

Most current BCIs use electroencephalography (EEG) as a tool to infer mental state and communicative intent (Lotte et al., 2007). EEG provides a low-resolution spatial map of electrical activity on the cortex (Niedermeyer and da Silva, 2005). Despite this low resolution, it has generally been favored for BCI applications due to its relatively simple setup and low cost. Some recent research has also investigated the usage of hemodynamic imaging technologies such as near-infrared spectroscopy (Sitaram et al., 2007) and transcranial Doppler ultrasound (Myrden et al., 2011), but these technologies cannot currently match the information transfer rate of EEG-BCIs. However, these hemodynamic imaging technologies may be useful in combination with EEG. Recent work on hybrid EEG-NIRS BCIs has shown that simultaneous measurement of electrical and hemodynamic activity on the cerebral cortex may allow for more accurate BCI operation by combining features from both modalities (Leamy et al., 2011; Fazli et al., 2012). More complex arrangements are also possible—Liu et al. (2012) have shown that attention measured based on NIRS may improve the reliability of an EEG-BCI, while Koo et al. (2015) showed that NIRS can be used to detect whether motor imagery has been performed while EEG is used to differentiate different types of motor imagery, allowing the development of a self-paced BCI.

EEG-BCIs that are used for communication and control typically employ one of two paradigms. The first depends upon involuntary neuronal reactions to presented stimuli, and has been described as a “reactive BCI” (Zander and Kothe, 2011). This includes BCIs that detect the P300 response to anticipated visual, auditory, or tactile stimuli (Hoffmann et al., 2008) and steady-state visually evoked potential (SSVEP) BCIs that detect the flicker frequency of the stimuli on which the user is fixated (Cheng et al., 2002). Both of these types of BCIs allow the user to choose one option from a grid of stimuli and are most commonly used as the basis for a spelling system. The second paradigm depends upon the detection of a voluntary cognitive activation, typically produced by performing a specific mental task. This has been described as an “active BCI” (Zander and Kothe, 2011). Active BCIs differentiate two or more mental tasks from each other, allowing the user to employ each task to communicate a different message. Differentiating more than two tasks from each other typically incurs a decrease in classification accuracy, and it is rare for more than four mental tasks to be used (Schlögl et al., 2005). Mental tasks used for active BCIs in previous research have included a rest state, motor imagery, mental arithmetic, and a verbal fluency task, among others (Pfurtscheller and Neuper, 2001; Curran and Stokes, 2003; Myrden et al., 2011).

One pervasive challenge in BCI research is the tendency for BCI accuracy to decrease over time due to the non-stationarity of the signals used (Shenoy et al., 2006). It is well-known that class distributions tend to change over time, and maintaining high BCI performance during long sessions and across weeks and months of usage is typically difficult (Shenoy et al., 2006). This inconsistent performance is a significant impediment to the adoption of BCIs as access modalities for individuals with disabilities and may also be a significant risk factor for the abandonment of BCIs by these individuals (Phillips and Zhao, 1993). It has been proposed that one cause of this inconsistent performance may be fluctuations in psychological variables such as alertness and distraction (Curran and Stokes, 2003; Millán et al., 2010). Systems that track this type of involuntary ongoing cognitive user state can be categorized as passive BCIs (Zander and Kothe, 2011). Examples include estimation of task engagement and attention (Berka et al., 2007; Ayaz et al., 2012; Hasenkamp et al., 2012; Harrivel et al., 2013), mental workload (Berka et al., 2007; Hirshfield et al., 2009), fatigue (Shen et al., 2008), and emotional state (Sitaram et al., 2011). These passive BCIs each use either EEG, NIRS, or fMRI, allowing them to be integrated with an active or reactive BCI that uses the same modality (or a complementary modality in the case of a hybrid BCI). Such a combination may allow adaptation to fluctuations in mental state, mitigating the observed variation in BCI performance over time. However, it is first imperative to verify that these fluctuations in mental state are related to variation in BCI performance, as to the best of our knowledge, this hypothesis has not been formally tested.

This paper investigates the effects of user mental state on BCI performance. Three mental states of particular interest were identified based on previous work—cognitive fatigue, frustation, and attention (Curran and Stokes, 2003). Subjective self-reported estimations of these three mental states were gathered from BCI users while playing a simple maze navigation game. These ratings were compared to BCI performance to identify relationships between mental state and classification accuracy. A multivariate analysis was also performed to identify a region in mental state space for optimal BCI performance. Finally, the class distributions of the rest and active tasks were analyzed in the feature space to determine the effects of changes in mental state on the individual signal features used for classification.

## 2. Materials and methods

### 2.1. Population

Twelve able-bodied participants (two male, average age 27.7 years) were drawn from graduate students and staff at Holland Bloorview Kids Rehabilitation Hospital. Participants had normal or corrected-to-normal vision and refrained from consuming caffeine for 4 h prior to each session. Participants provided written informed consent, and the experimental protocol was approved by the Holland Bloorview Research Ethics Board.

### 2.2. Instrumentation

During each session, electrical signals from the cortex were recorded using a B-Alert X24 wireless EEG headset (Advanced Brain Monitoring, Carlsbad, CA, USA). Signals were recorded from the Fz, F1, F2, F3, F4, Cz, C1, C2, C3, C4, CPz, Pz, P1, P2, and POz locations according to the international 10-20 system (Homan et al., 1987). Signals were band-pass filtered between 2 and 30 Hz, and artifacts resulting from eye movements were removed using independent component analysis (Mognon et al., 2011).

### 2.3. Training sessions

Participants completed two training sessions on separate days. The goal of these sessions was to identify a mental task that could reliably be differentiated from rest for each participant. Four candidate mental tasks were considered—mental arithmetic, motor imagery, music imagery, and word generation. Multiple tasks were considered because the most effective BCI task typically varies across participants (Guger et al., 2003). The mental arithmetic task required participants to perform a repeated addition or subtraction task in their head. The motor imagery task required participants to imagine performing finger-to-thumb opposition with the hand of their choice. The music imagery task required participants to sing a song of their choice in their heads. The word generation task required participants to silently think of as many words as possible that began with a given letter.

During each training session, participants completed 150 trials. These were evenly divided between the four candidate mental tasks and a rest task, during which participants were instructed to relax and let their minds wander. Each trial was 15 s long, consisting of a 5-s preparation period during which a visual cue was displayed to indicate the required task for the trial; a 5-s task period during which the participant performed the required task; and a 5-s cool-down period before the next trial began. Participants were instructed to remain silent and still during the preparation and task periods to minimize motor artifacts. The visual cue for each trial was displayed on a computer monitor using a custom LabVIEW interface. To avoid mislabeling training data, participants were prompted to report whether they had successfully completed each trial at the end of the cool-down period. This was done through an on-screen dialog box that had to be completed before the next trial could begin. At the end of each training session, participants ranked the four candidate mental tasks in order of preference for future BCI usage.

### 2.4. BCI development

Following the completion of the training sessions, a BCI was trained to differentiate each candidate mental task from the rest task. There were a total of 60 trials for each task. For each signal from each electrode, the spectral power within the signal in 1-Hz increments (from 0–1 to 29–30 Hz) was estimated by summing the squares of the corresponding Fourier coefficients. These local spectral power estimates yielded 450 individual features (30 frequencies from 15 electrodes) for each trial.

Two feature selection methods were used to reduce the dimensionality of the feature set to between 1 and 12 features for classification. In the first method, a fast correlation-based filter (FCBF) directly reduced the dimensionality from 450 to the target number of features. This resulted in most of the feature set being discarded. In the second, this 450-dimensional feature set was reduced by clustering highly correlated features and performing principal component analysis (PCA) on each cluster to compute 75 intermediate features before using a FCBF to arrive at the target number of features. The latter approach was included to accommodate tasks that elicited widely distributed cortical activation at varying frequencies.

For each feature space dimensionality, a linear discriminant analysis (LDA) classifier (Bishop, 2006) was trained for each candidate task and feature selection method. Ten runs of ten-fold cross-validation were performed and the average classification accuracy across the folds was computed. This resulted in a set of 24 different classifiers for each task. The classifier that yielded the highest classification accuracy was identified for each task and the tasks were then ranked by their maximum accuracy. Participants were presented with these accuracy-based task rankings along with their own rankings of task preference. Based on this information, they were allowed to choose which active task they wanted to use for the remainder of the study.

### 2.5. Testing sessions

Participants completed three testing sessions on separate days. During these sessions, they used a BCI based on the task that they selected at the end of the training sessions to play a simple maze navigation game that was programmed in LabVIEW. Participants attempted to complete a series of 10 mazes. Each session started with the first (and simplest) maze. Mazes grew more difficult as the session progressed, but this was primarily due to the number of intersections between the origin and destination rather than the cognitive difficulty of plotting a path through the maze.

**Figure 1:**
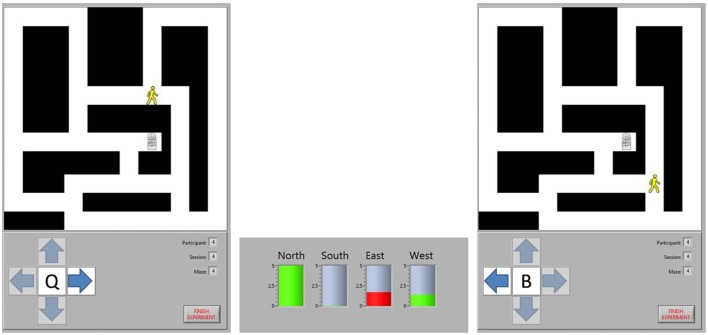
**Maze navigation**. The position of the participant is represented by the image of a person and the destination by the image of a door. The participant chose to move east at Intersection 1, so they are shown a cue for the word generation task when the arrow pointing east is highlighted. A cue for the rest task was shown when the north and west arrows were highlighted. The BCI analyzed the task period from each direction, predicting that the north and west task periods represented rest tasks (green bars) while the east task represented the word generation task (red bar). Consequently, the BCI moved the participant to Intersection 2, ignoring the intermediate intersection where the only options would have been to continue moving in the same direction or to go back to the last intersection. At the new intersection, the participant chose to move west to continue approaching the exit, so a new word generation cue was shown when that direction was highlighted.

Participants navigated through the maze by moving from intersection to intersection. Their current position was indicated by an image of a person, while the destination was indicated by an image of a door. At each intersection, there were between two and four potential directions (labeled as north, south, east, and west) in which movement to another intersection was possible. Participants were prompted to select the direction in which they wanted to travel from an on-screen window. Subsequently, the potential navigational directions were highlighted one at a time for 5 s each, constituting four task periods. Each task period was punctuated with a 5-s break. When a direction was highlighted, appropriate task cues were shown, namely the cue for the active task for the selected direction of travel and the cue for the rest task for all other directions. The selected direction of travel was recorded only to label data for future analysis and to ensure that appropriate task cues were presented during each task period. An example of the game, depicting an initial intersection, a BCI decision, and the subsequent intersection, is shown in Figure 1.

The EEG recording from each 5-s task period was classified in real-time by the BCI. When the task period had been completed for each potential direction, the BCI decided which task period was most likely to represent the active task rather than the rest task, and the image on screen was moved in the corresponding direction. However, before this movement was displayed, the participant was prompted to self-report their perceived levels of fatigue, frustration, and attention. Each of these mental states was rated by moving a slider on a continuous scale from 0 to 1 with textual anchors at either end (e.g., “Least fatigued” and “Most fatigued”). A new maze was automatically loaded when the current maze was completed (i.e., when the participant navigated to the door) and the session was terminated either after completion of the tenth maze or once 50 min had elapsed.

## 3. Results

### 3.1. Choice of BCI task

Offline performance for each participant during the training sessions is summarized in Table 1. Word generation was selected as the optimal BCI task by nine participants, motor imagery by two participants, and music imagery by the final participant. The average classification accuracy of the selected task was 72.4%. This exceeded the minimal BCI performance criteria of 70% despite the short task duration and relatively small selection of electrodes. Since the analysis focused on the effects of mental state on BCI performance, it was not necessary to obtain extremely high accuracy. In fact, high accuracy may have inhibited the analysis, as it is likely that a smaller range of ratings would be induced for each mental state if BCI performance was close to perfect.

**Table 1:** **Classification accuracies for each participant during the training sessions for the mental arithmetic (MA), motor imagery (MI), music imagery (MuI), and word generation (WG) tasks**.

**Participant**	**MA%**	**MI%**	**MuI%**	**WG%**	**Selected task**
1	87	95	67	74	MI
2	63	60	63	70	WG
3	58	75	68	50	MI
4	65	64	66	70	WG
5	80	77	62	82	WG
6	62	55	57	60	WG
7	N/A	69	61	69	WG
8	50	71	65	71	WG
9	56	61	59	66	WG
10	82	81	73	83	WG
11	69	N/A	71	64	MuI
12	66	55	56	57	WG

*Word generation was selected by nine participants, motor imagery by two participants, and music imagery by the remaining participant. Entries labeled as “N/A” indicate occasions when a mental task was removed from the second training session for a participant due to both poor classification accuracy during the first training session and placement as the least preferred task for that participant following the first training session*.

### 3.2. Online BCI performance

Participant 7 was excluded from this analysis as he or she was not able to control the BCI during the testing sessions, and Participant 8 was unable to attend the testing sessions. One testing session for each of Participants 2 and 3 could not be analyzed due to signal quality issues. Three testing sessions were analyzed for all other participants. Although retraining the BCI after each testing session for all participants would have resulted in higher accuracies, it was avoided to minimize the number of factors affecting classification accuracy. Consequently, BCIs were retrained after testing sessions only when the experimenters felt it absolutely necessary in order to maintain motivation for participants. This occurred only for the final testing sessions for Participants 11 and 12. All other participants used the same BCI for all testing sessions.

Two metrics were considered: the balanced individual classification accuracy, referring simply to the average of sensitivity and specificity for the individual tasks; and the collective classification accuracy, referring to the proportion of maze intersections at which the BCI correctly identified the intended direction of transit. There were typically three or four potential directions of transit, so the collective accuracy was expected to be lower than the individual accuracy.

**Figure 2:**
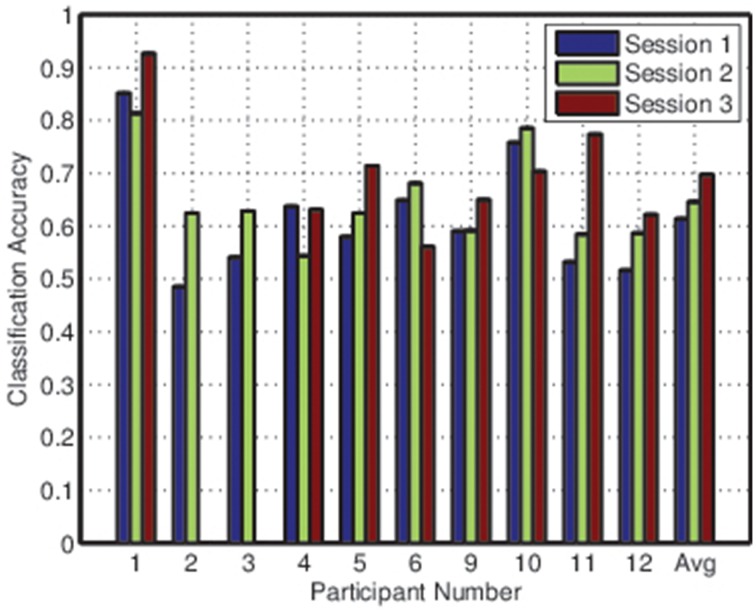
**Balanced individual classification accuracies for all participants during each session**.

**Figure 3:**
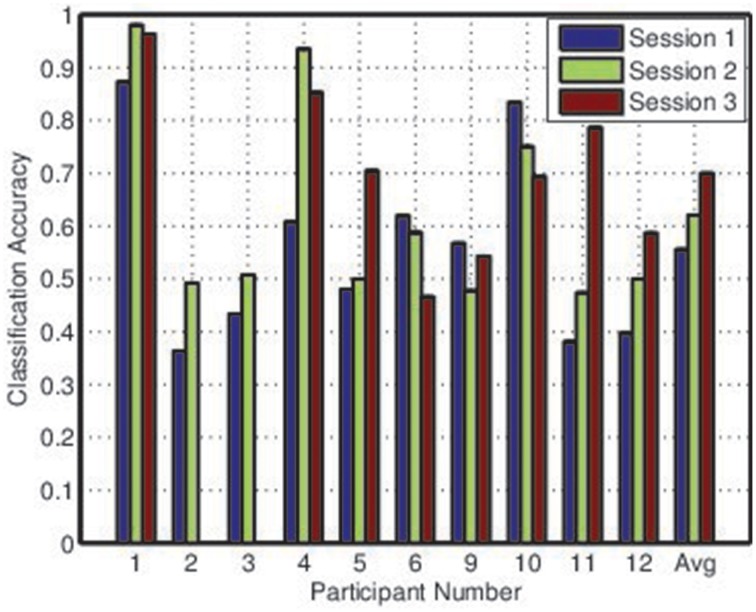
**Collective classification accuracies for all participants during each session**.

Figures 2, 3 depict the individual and collective accuracies, respectively, for each participant during each online session. Despite the non-adaptive classifier, four of ten participants exceeded the 70% threshold for individual classification accuracy during one or more testing sessions. Furthermore, five of ten participants achieved a collective classification accuracy that exceeded this threshold during one or more sessions.

Both individual and collective classification accuracy increased in the second and third sessions, suggesting that participants became more proficient with the BCI over time. For the third session, both individual and collective classification accuracy neared the 70% threshold when averaged across all participants.

### 3.3. Effects of fatigue, frustration, and attention on BCI performance

For each participant, self-reported ratings for each mental state during each session were quantized to two levels, defined as, for example, “low fatigue” and “high fatigue.” The cut point for quantization was varied for each participant and mental state to ensure that each level contained as close to the same amount of trials as possible. Each individual trial was categorized as either low or high for each mental state and the classification accuracy at each level across all participants was computed. By ensuring that each session was equally represented in the low and high categories for each mental state, this approach controlled for learning effects. These results are presented in Table 2 for the individual classification accuracy.

**Table 2:** **Individual classification accuracies at low and high levels for each mental state**.

**Level**	**Fatigue**	**Frustration**	**Attention**
Low	62.6	61.7	63.8
High	65.0	65.7	64.2

*Classification accuracies were based on 4814 trials across ten participants, and each level contained roughly the same number of trials*.

Classification accuracy at low fatigue was about 2.5% inferior when compared to high fatigue, a result that approached significance using the chi-squared test (*p* = 0.088). The 4% difference in classification accuracy between low and high frustration was statistically significant (*p* = 0.0038). There was no significant difference between classification accuracy at low attention and high attention levels. However, these findings collectively indicate that there is a significant relationship between BCI performance and mental state.

To investigate whether the choice of mental task influenced the relationship between mental state and BCI performance, participants were split into two categories—those who chose word generation as the active task and those who did not—and the preceding analysis was repeated. The results for each group are depicted in Table 3. While frustration appeared to affect the two groups similarly, fatigue had more impact on the WG group and attention on the not-WG group. However, due to the small sample sizes incurred by splitting the group in two, further research with control groups of equal size for each task would be necessary to draw significant conclusions.

**Table 3:** **Classification accuracies at low and high levels for each mental state**.

**Task**	**Level**	**Fatigue**	**Frustration**	**Attention**
WG	Low	61.5	60.7	62.3
	High	64.0	64.4	63.3
Not WG	Low	66.7	64.3	68.8
	High	67.2	68.8	66.0

*Classification accuracies were based on 4814 trials across ten participants, and each level contained roughly the same number of trials*.

Since the two-level quantization of each rating was a simplistic means of investigating these effects, further analysis was conducted using normalized values for each mental state. For each session, the self-reported ratings for each mental state were normalized to zero mean and unit variance. For each mental state, all trials across all participants were then sorted by their normalized rating. Since each trial was also associated with a classification result (i.e., either a correct or incorrect decision by the BCI), this allowed the construction of a binary sequence representing BCI performance over the full range of normalized ratings. This sequence was smoothed to minimize the noise produced by the usage of individual classification results, resulting in a classification accuracy curve *C*_*actual*_ for each state.

**Figure 4:**
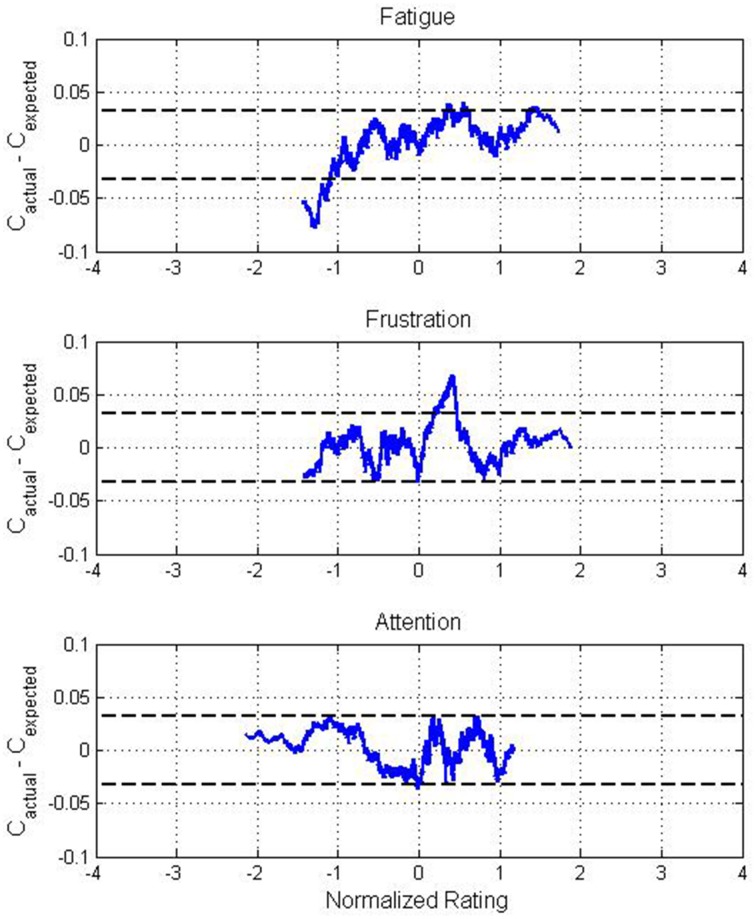
**Difference between actual and expected classification accuracy across all participants for normalized values of self-reported fatigue, frustration, and attention ratings**. The distributions of fatigue and frustration ratings were positively skewed while the distribution of attention ratings was negatively skewed, causing the apparent variation in range between states. Dotted lines represent the 90% confidence interval for the mean of the deviation between actual and expected classification accuracy.

Since ratings from each participants were not uniformly distributed within the range of ratings for each state, *C*_*actual*_ was biased due to individual variations in classification accuracy. To mitigate this, an expected classification accuracy curve *C*_*expected*_ was constructed by replacing the actual classification result from each trial with the average classification accuracy from the session within which each trial originated. The same smoothing process was performed, and the effects of mental state on BCI performance were assessed based on the difference between *C*_*actual*_ and *C*_*expected*_, as depicted in Figure 4. Through random sampling, the difference between actual and expected classification accuracy was observed to follow a normal distribution with a 90% confidence interval of 0 ± 0.032. The bounds of this confidence interval are depicted in Figure 4.

These figures support the results from Table 2 while also providing a higher-resolution view of the trends in classification accuracy. Performance was poor for low levels of self-reported fatigue, with the difference between actual and expected accuracy surpassing −7%, well outside of the 90% confidence interval. For frustration, higher classification accuracies than expected were exhibited for moderate values, peaking at approximately +7%. In contrast, the difference between actual and expected classification accuracy over the full range of attention ratings was small, remaining almost entirely within the 90% confidence interval.

### 3.4. Multivariate analysis

**Figure 5:**
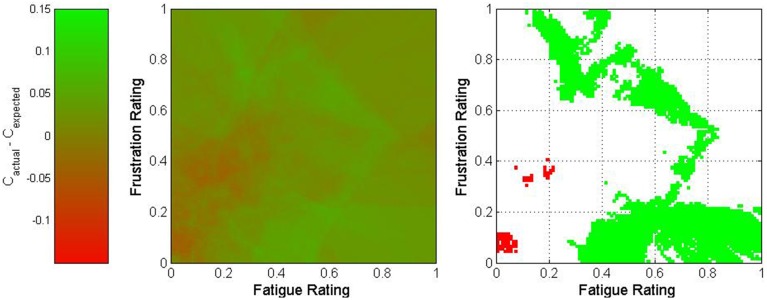
**Two-dimensional view of the difference between actual and expected classification accuracies as a function of fatigue and frustration**. The middle graph depicts the variation in classification accuracy as shown on the legend on the left, and the right graph shows only regions for which the difference exceeded 3.2% (in green) or was less than −3.2% (in red).

**Figure 6:**
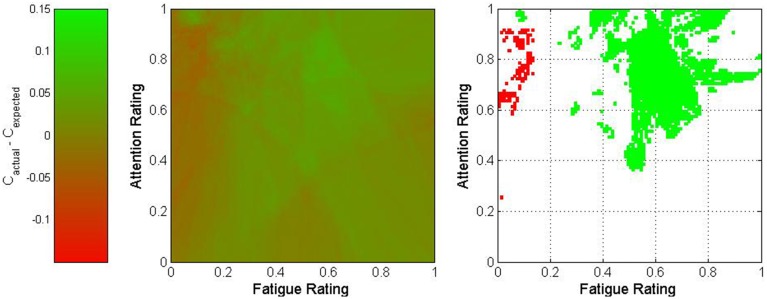
**Two-dimensional view of the difference between actual and expected classification accuracies as a function of fatigue and attention**. The middle graph depicts the variation in classification accuracy as shown on the legend on the left, and the right graph shows only regions for which the difference exceeded 3.2% (in green) or was less than −3.2% (in red).

**Figure 7:**
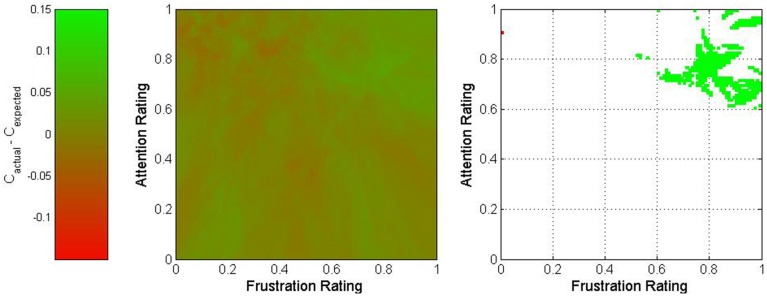
**Two-dimensional view of the difference between actual and expected classification accuracies as a function of frustration and attention**. The middle graph depicts the variation in classification accuracy as shown on the legend on the left, and the right graph shows only regions for which the difference exceeded 3.2% (in green) or was less than −3.2% (in red).

The previous analysis considered only the effects of each individual mental state. A multivariate analysis of the effects of mental state on BCI performance was also conducted by analyzing the effects of each combination of two states. The raw self-reported ratings were extracted for each participant without quantization. A grid was constructed across the full range from 0 to 1 for each mental state with a resolution of 0.01. At each point within the grid, the nearest 500 trials, regardless of participant, were identified. For this set of 500 trials, the actual classification accuracy *C*_*actual*_ was computed. In addition, the expected classification accuracy was computed as:

(1) $$C_{expected}=\Sigma_{p}w_{p}C_{p,s}$$

where *w*_*p*_ represents the proportion of the nearest 500 trials which originated from Participant p and *C*_*p,s*_ represents the overall classification accuracy of all trials originating from session s for Participant p. The difference between actual and expected classification accuracies was used to characterize this point. The results of this process are depicted in Figures 5–7 for fatigue and frustration; fatigue and attention; and frustration and attention, respectively. Again, the difference between actual and expected classification accuracy was compared to the 90% confidence interval, established previously as 0 ± 0.032.

The fatigue-frustration and fatigue-attention graphs reveal relatively contiguous optimal regions for BCI control. For fatigue-frustration, two optimal regions are apparent—one from moderate to high fatigue and low to moderate frustration and one from low to moderate fatigue and moderate to high frustration. Of these, the former is larger and more consistent across a wide region in mental state space. For fatigue-attention, there is an optimal region for moderate to high fatigue and attention. Again, these findings corroborate the univariate analyses despite the usage of raw ratings rather than quantized or normalized ratings.

The graph for frustration-attention is more equivocal. The area within mental state space that exceeded the bounds of the 90% confidence interval was small and located in the high frustration and high attention region. This may imply that high attention is necessary to compensate for high frustration. However, given the small size of this region, this could also potentially be a result of random variation.

### 3.5. Effects of mental state on class distributions

**Figure 8:**
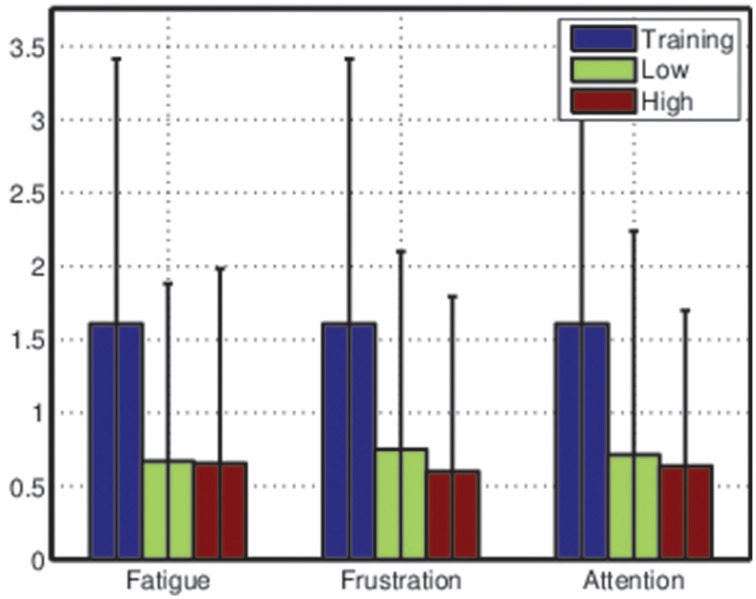
**Average Fisher scores across all participants during training sessions and testing sessions**. Data from the testing sessions were split into low and high categories for each mental state before the Fisher score was computed. Error bars are extremely wide due to large variance in class separability (and thus classification accuracy) between participants—see Table 1.

The effects of mental state on BCI performance were also analyzed from a signal feature perspective. As in the univariate analyses, all trials for each participant were split into low and high categories for each mental state. In this case, the LDA classifier trained for each participant after the training sessions was used to project each trial to one dimension. The separability of the rest and active tasks were then estimated by computing the Fisher score for each projection. The Fisher score is defined as (Foley and Sammon, 1975):

(2) $$J=\frac{|\mu_{1}-\mu_{2}{|}^{2}}{s_{1}^{2}+s_{2}^{2}}$$

where μ_1_ and *s*_1_ represent the mean and the variance of the projected values for the rest class and μ_2_ and *s*_2_ represent the mean and the variance of the projected values for the active class. The results of this analysis are depicted in Figure 8.

These results suggests that the classifiers trained based on the training sessions were much less effective during the testing sessions, accentuating the importance of frequent retraining. However, the average differences between Fisher scores for low and high ratings for each mental state also suggest that, for some participants, mental state may affect class distributions in feature space.

**Figure 9:**
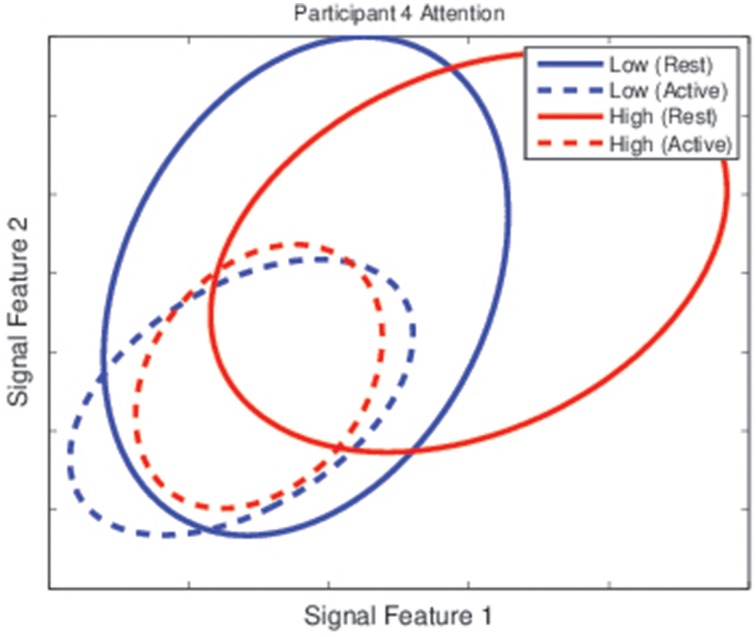
**Class distributions for the rest and active task for Participant 4 during the testing sessions**. Each ellipse represents the distribution of one class (the rest task for solid lines and the active task for dashed lines) under one categorization of attention levels (low in blue lines, high in red lines). While classes were nearly separable when attention was high, they were unseparable when attention was low.

To verify this, the class distributions for the rest and active tasks under different mental state conditions were inspected based on two of the features used for classification. Figure 9 shows the class distributions of each task for Participant 4 under low and high attention conditions. The center of each ellipse represents the class mean under that condition while the size of the ellipse represents the 67% confidence interval for the class, oriented along the eigenvectors of the covariance matrix. Even in this low-dimensional space (the classifier for this participant used 10 features), it is evident that modulations in mental state affect class distributions, as the two classes are nearly separable when attention is high but inseparable when attention is low.

## 4. Discussion

### 4.1. Optimal mental state for BCI control

It is clear from our univariate analyses that mental state and BCI performance are closely intertwined. However, some of our observations were surprising. Online BCI performance was significantly less accurate during the trials for which participants reported the lowest fatigue levels and significantly more accurate during the trials for which participants reported high frustration levels. This was observed when each trial was categorized by quantized ratings for each state and also when ratings were normalized within each session. Based on the same analyses, attention seemed to have little impact on BCI performance. Analysis based on the choice of active task suggests that there may be task-related effects, but further investigation would be necessary for statistical verification.

Self-reported mental state ratings were quantized and normalized for these initial analyses in an attempt to account for the fact that different participants may have anchored their ratings differently on the continuous scales used for each state. However, one shortcoming of this approach was that it ignored differences in average mental state. Since one participant reporting a higher average fatigue level than another could be either a result of variation in anchoring or a legitimate difference in fatigue levels, the raw ratings were used for the multivariate analysis in order to compare the results.

This multivariate analysis suggested the presence of optimal mental state regions for BCI control. The most interesting observation came from the fatigue-attention analysis, which showed that the highest accuracies occurred when moderate values were maintained for fatigue and moderate to high values for attention. BCI performance decreased markedly when these states varied, particularly for low fatigue and high attention. The multivariate analysis also presented a more nuanced portrait of the effects of each mental state on BCI perfomance. The fatigue-frustration and frustration-attention cases both showed interactions between pairs of states. In general, the former analysis showed that optimal performance occurred for either moderate fatigue and high frustration or high frustration and low fatigue. In contrast, the low fatigue and low frustration case exhibited notably poor performance.

Although there is no pre-existing BCI literature for comparison, some support for these results can be found in other disciplines. The state of psychological flow has been identified as a requirement for excellent performance in many fields (Jackson et al., 2001; Demerouti, 2006; de Manzano et al., 2010). Flow is characterized by what Romero describes as effortless attention, a state of deep concentration where perceived effort is generally lower than would be expected (Romero and Calvillo-Gámez, 2014). This is contrasted with effortful attention, in which the perceived effort to achieve focus is quite high and individuals must fight to maintain deep concentration. We hypothesize that, due to the high perceived effort, effortful attention is likely to be characterized by higher self-reported fatigue and potentially higher self-reported attention than effortless concentration. Since optimal performance can be expected during effortless attention, this could produce the pattern seen in Figure 6.

The role of frustration has also attracted attention in previous research. In learning studies, it has been observed that frustration, in moderation, is not necessarily a negative factor (Baker et al., 2010). In fact, the presence of frustration during a difficult task may simply represent motivation, which is a factor likely to improve performance. However, studies have also observed that high frustration induces boredom, reducing attention and leading to poor performance (D'Mello and Graesser, 2012). This implies that optimal performance may be associated with moderate frustration, corroborating our findings, particularly those depicted in Figure 4.

There is one important caveat regarding this study. It has been shown that fluctuations in mental state are related to fluctuations in BCI performance. However, it stands to reason that since these fluctuations affect the underlying class distributions of the active and rest tasks, as seen in Figure 9, the classifiers used for each BCI were dependent upon the mental state experienced by each participant during the training sessions. Consequently, it may be that the optimal mental state for BCI control is simply that which most closely approximates the mental state from the training sessions. However, given the length of each training session, the commensurate unlikelihood that mental state was consistent throughout, and the unusual topography of the optimal mental state regions in Figures 5–7, it is more likely that the results were affected by both the mental state during the training sessions and the inherent superiority of certain psychological conditions for BCI control. Regardless of which factor is most responsible for the relationship between mental state and BCI performance, these results strongly suggest that such a relationship does exist. This motivates future investigation of psychologically adaptive BCIs.

### 4.2. Toward psychologically adaptive BCIs

It has been proposed that there are two ways in which a computer system can adapt to information regarding the cognitive state of a user. These are overt adaptation, in which the adaptation is apparent to the user, and covert adaptation, in which it is not apparent to the user (Fairclough, 2009). These definitions can also be applied to the design of psychologically adaptive BCIs.

Overt adaptation, although potentially more effective than covert adaptation for modifying user state, also has a potentially higher cost (Fairclough, 2009). For a BCI, overt adaptation would require an attempt to modify user mental state to bring it closer to the optimal region, likely taking the form of an adaptive user interface (Tan and Nijholt, 2010). Such an interface could use targeted stimuli or helpful feedback to mitigate undesirable changes in mental state (Fairclough, 2009; Tan and Nijholt, 2010). The interface could also take more drastic steps, potentially going so far as to automatically deactivate the BCI when extremely low attention is detected, reactivating only when the user's attention has returned. The interface could even modify the timing variables of the BCI, extending task durations when it is likely that classification will be inaccurate, a psychologically-driven approach with some similarities to the evidence accumulation algorithms that are often used for online classification. The danger of overt adaptation lies in the potential for false alarms (Fairclough, 2009). Explicit interventions that are not required may actually further inhibit BCI control by inducing additional frustration or distraction. It may be wise to use overt adaptation sparingly (Fairclough, 2009).

Covert adaptation, on the other hand, could involve modifications to the classifier itself. There are several potential methods by which this could be implemented. First, we observed in Figures 2, 3 that there was little difference between individual and collective classification accuracy even though the individual accuracy was based on a binary decision and the collective accuracy on a decision that typically involved three or four options. This implies, for the LDA classifiers that were used, that there was more difficulty locating an appropriate value for the bias parameter than for the weight vector. Thus, an adaptive bias parameter based on mental state may allow for covert adaptation without repeated classifier retraining. Second, given the effects of mental state on class distribution observed in Figure 9, it is possible that selective online resampling of the training set and retraining of a simple classifier (such as LDA) could be implemented. This would require online estimation of user mental state, the selection of the training points most closely matching this current mental state, and the training of a classifier based on this subset of the training data. Since these adaptations would go unnoticed by the BCI user, they could be employed as frequently as necessary (Fairclough, 2009).

There are two significant limitations for any psychologically adaptive BCI. First, it is obviously necessary to achieve accurate detection of these changes in mental state. Our group is currently working on achieving reliable differentiation between low and high values of the three mental states in question. Second, any psychological adaptation that is implemented must be adaptive in itself. Significant differences were observed across participants in terms of the reactivity of BCI performance to changes in mental state, and it is unlikely that a “one size fits all” approach will be sufficient.

## 5. Conclusions

In this study, we investigated the effects of mental state on BCI performance. We observed that the relationships between these variables were complex, rather than monotonic. There appear to be optimal operating conditions where fatigue, frustration, and attention levels are most appropriate for effective control of an EEG-BCI. Moreover, signal features are affected by changes in mental state, potentially necessitating classifier adaptation. Future work should consider the development of BCIs that display both overt adaptation to keep user mental state within the optimal region and covert adaptation that automatically modifies the BCI classification algorithm to adapt to changes in mental state. This will allow the development of BCIs that are more robust to changes in mental state.

### Conflict of interest statement

The authors declare that the research was conducted in the absence of any commercial or financial relationships that could be construed as a potential conflict of interest.
